# Current Diagnosis and Treatment Options for Cutaneous Adnexal Neoplasms with Follicular Differentiation

**DOI:** 10.3390/ijms22094759

**Published:** 2021-04-30

**Authors:** Iga Płachta, Marcin Kleibert, Anna M. Czarnecka, Mateusz Spałek, Anna Szumera-Ciećkiewicz, Piotr Rutkowski

**Affiliations:** 1 Department of Soft Tissue/Bone Sarcoma and Melanoma, Maria Sklodowska-Curie National Research Institute of Oncology, 02-781 Warsaw, Poland; iplachta@outlook.com (I.P.); marcin.kleibert@interia.pl (M.K.); mateusz@spalek.co (M.S.); piotr.rutkowski@pib-nio.pl (P.R.); 2 Faculty of Medicine, Medical University of Warsaw, 02-091 Warsaw, Poland; 3 Department of Pathology and Laboratory Diagnostics, Maria Sklodowska-Curie National Research Institute of Oncology, 02-781 Warsaw, Poland; szumann@gmail.com; 4 Department of Diagnostic Hematology, Institute of Hematology and Transfusion Medicine, 00-791 Warsaw, Poland

**Keywords:** follicular tissue, adnexal, tumors, neoplasm, treatment, diagnosis

## Abstract

Neoplasms derived from follicular tissue are extremely rare. Clinically, they are reported as non-symptomatic, slow-growing nodules. These lesions are mainly benign, but the malignant type can occur. Mainly middle-aged people (50–60 years of age) are affected. These carcinomas are mainly localized on the head and neck or torso. They can be locally aggressive and infiltrate surrounding tissue and metastasize to regional lymph nodes. In the minority of cases, distant metastases are diagnosed. Quick and relevant diagnosis is the basis of a treatment for all types of tumors. The patient’s life expectancy depends on multiple prognostic factors, including the primary tumor size and its mitotic count. Patients should be referred to a specialized skin cancer center to receive optimal multidisciplinary treatment. This article tries to summarize all the information that is currently available about pathogenesis, diagnosis, and treatment methods of follicular tumors.

## 1. Introduction

**Figure 1:**
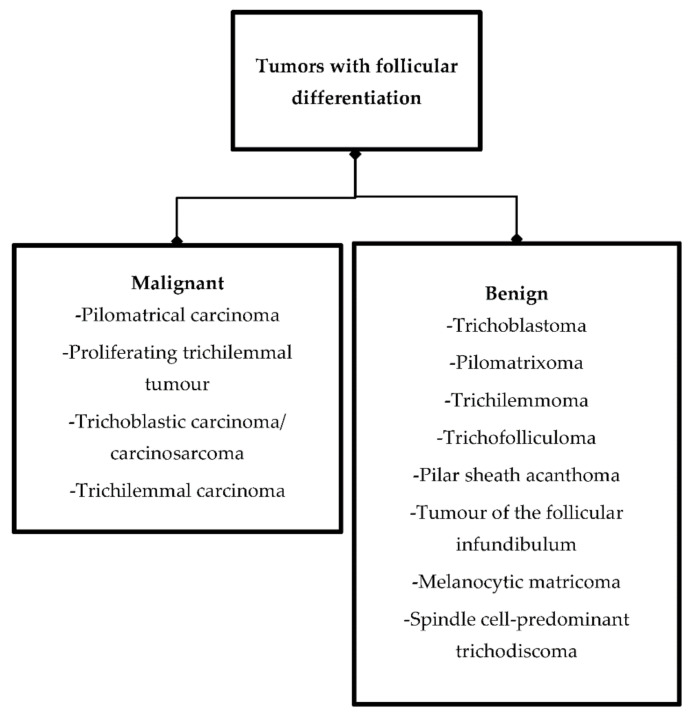
Division of tumors with follicular differentiation.

Cutaneous adnexal tumors are an extensive group of lesions divided into a few categories depending on morphological differentiation. They can derive from apocrine, eccrine, sebaceous glands, or follicles [1]; some of these neoplasms display differentiation toward more than one cell lineage, making their precise classification difficult. This article focuses on tumors with follicular differentiation, which are the large and heterogeneous group (Figure 1).

**Figure 2:**
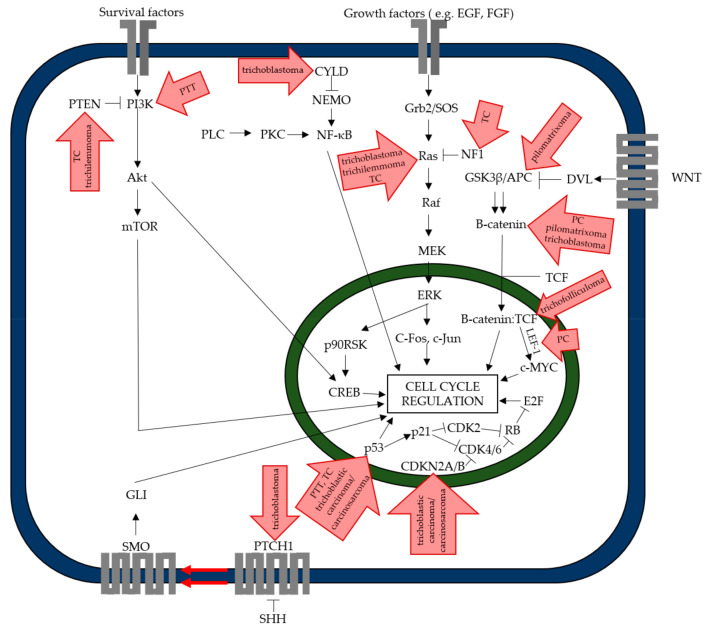
Disturbance of intracellular signaling pathways. Single arrow—activation; double arrow—activation by inhibition; inhibition arc—inhibition. Akt—protein kinase B; APC—adenomatous polyposis coli; CDK—cyclin-dependent kinase; CDKN—cyclin-dependent kinase inhibitor; CREB—cAMP response element-binding protein; CYLD—ubiquitin carboxyl-terminal hydrolase; DVL—dishevelled; ERK—extracellular signal-regulated kinase; EGF—epidermal growth factor; FGF—fibroblast growth factor; GLI—glioma-associated oncogene; Grb—growth factor receptor-bound protein; GSK—glycogen synthase kinase; LEF-1—lymphoid enhancer-binding factor-1; MEK—mitogen-activated protein kinase kinase; mTOR-mechanistic target of rapamycin; NEMO-NF-Kappa-B essential modulator; NF-κB-nuclear factor kappa-light-chain-enhancer of activated B cells; p90RSK—90 kDa ribosomal s6 kinases; PI3K-phosphoinositide 3-kinase; PKC-protein kinase C; PLC-phospholipase C; PTCH1—patched 1; PTEN—phosphatase and tensin homolog; RB—retinoblastoma protein; SHH—sonic hedgehog; SMO—smoothened; SOS—Son of Sevenless; TCF—transcription factor.

Most of these neoplasms develop *de novo* from one part of the hair, such as the bulb, the isthmus, or the infundibulum. The following lesions exhibit features suggestive of differentiation towards or derivation from the hair germ or matrix [2]. The majority of follicular neoplasms can occur as sporadic cases, but some inherited syndromes such as Brooke–Spiegler (autosomal dominant disease (AD), the mutation is in *CYLD* (CYLD Lysine 63 Deubiquitinase) suppressor gene), Gardner’s syndrome (AD, the mutation in *APC* (adenomatous polyposis coli) suppressor gene), Cowden syndrome (AD, germline mutation in *PTEN* (phosphatase and tensin homolog) suppressor gene), and Rombo syndrome (unknown etiology) can predispose people to them [3,4,5,6,7]. Moreover, in some cases, a relationship with viral infection is suggested [8]. Due to the fact that these lesions are rare, the driver mutations are unknown, with several exceptions presented below in the table (Table 1, Figure 2).

## 2. Tumors with Follicular Differentiation

### 2.1. Malignant Tumors with Follicular Differentiation

#### 2.1.1. Pilomatrical Carcinoma

Pilomatrical carcinoma (PC) is a malignant appendageal tumor derived from follicular tissue. In one study, authors assess that caudal-related homeobox transcription factor 2 (CDX2) and lymphoid enhancer-binding factor 1 (LEF-1) can be a possible regulator and marker of pilomatrical carcinoma development. CDX2 and LEF-1 performed at least as well as β-catenin 1, in which the mutation is most often described (*CTNNB1*) (Table 1) [9].

Pilomatrical carcinoma is a rare skin cancer. Less than 150 cases have been reported. Male predominance in the incidence is observed (approximately 3:1 M (male): F (female)). These carcinomas usually occur in middle-aged or elderly white (~81%) adults (mean age 52 years old) [24,25].

**Figure 3:**
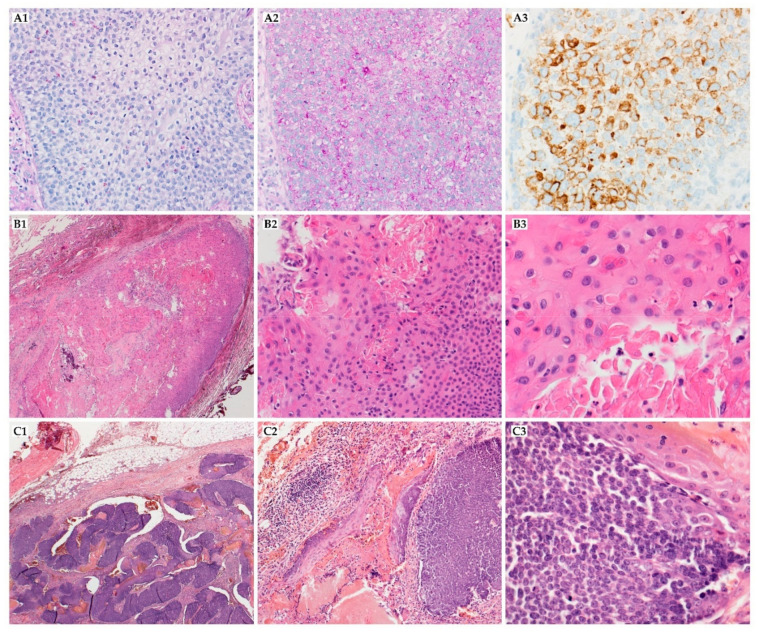
Malignant tumors with follicular differentiation. (**A1**–**A3**) trichilemmal carcinoma, composed of atypical cells with clear cells with sharply defined borders, prominent nucleoli, and frequent mitoses and [(**A1**) (400×, HE)], PAS-positive cytoplasm [(**A2**) (400×, PAS)], it shows CK14 expression, which is characteristic for follicular infundibulum differentiation (A3); (**B1**–**B3**) proliferating trichilemmal tumor, well-circumscribed nodule with trichilemmal keratinization without granular layer [(**B1**) (20×, HE), (**B2**) (200×, HE) and (**B3**) (400×, HE)]; (**C1**–**C3**) pilomatrical carcinoma, poorly circumscribed tumor with deep dermis invasion fields [(**C1**) (20×, HE)], solid aggregates of immature basaloid cells with less abundant shadow cells [(**C2**) (100×, HE)], neoplastic cells have scant cytoplasm, vesicular nuclei and high mitotic activity [(**C3**), (200×)].

The majority of PCs occur on the head (43%), neck (15%), back (11%), upper extremity (11%), lower extremity (11%), chest (6%), buttocks (2%), inguinal region (2%), and axilla (1%) [25]. It presents as a solitary nodule with frequent ulceration of the epidermal surface. Tumors vary in size, from 1–20 cm (mean = 4.6 cm). The reported average period between first consultation and diagnostic biopsy was 44.8 months. PC must always be considered in the differential diagnosis of the head-and-neck region’s hard solitary tumors due to high recurrence rate after simple excision. On the microscopic level, the lesion is composed of solid aggregates of immature basaloid cells. Neoplastic matrical cells show scant cytoplasm and other malignant features (Figure 3(C1–C3)). The shadow cells are very characteristic of pilomatricoma and PC.** However, their presence is much less marked on the PC. In some cases, rapid tumor progression can be observed [1,26]. Beta-catenin is expressed in the basaloid cells’ cytoplasm and nucleus, but BerEP4 (antibody to epithelial cell adhesion molecule/EPCAM) is described as negative [1].

**Figure 4:**
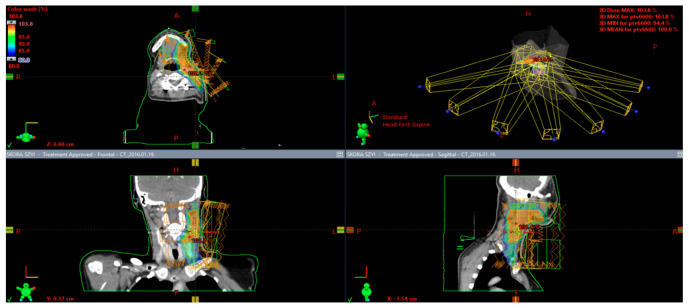
Adjuvant radiotherapy after surgical excision with positive microscopical margins of pilomatrix carcinoma localized on the cervical skin. The intensity-modulated radiotherapy technique with simultaneous integrated boost was used. The patient received 59.4 Gy in 1.8 Gy fractions for regional lymph nodes and 66 Gy in 2 Gy fractions for high-risk clinical target volume that covered tumor bed and surrounding skin.

The most widely-reported treatment is wide surgical excision (WLE), but Mohs micrographic surgery (MMS) is reported to be effective [24,27]. The wide excision definition is not clear and varies with the margin from 5 mm to 3 cm in the literature. Initially, most lesions are treated with simple excision, but the cases of local recurrence following simple excision (~83%, an average of 11.6 months) even with tumor-free surgical margins have been reported; because of it, the long-term follow and WLE (recurrence rate = 23%, an average of 6.9 months) are recommended [28]. However, WLE is not superior in preventing metastasizing in comparison with simple excision. The main predictors of metastases are local recurrence (HR (hazard ratio) = 3.45, *p* < 0.0413) and primary location on the extremities. Lymph node and systemic metastases were observed in ~13% of the reported cases on average after 21.4 months [25]. In the literature, only one case was reported with the margin greater than 1 cm, which develops metastasis [17]. Radiation and chemotherapy have been used with mixed effect for palliation of metastatic. All reported cases confirmed that PC has primary resistance to the utilized cytotoxic or immunomodulating agents (bleomycin, 5-fluorouracil, cisplatin, vinblastine, interferon, paclitaxel) [29]. Radiotherapy (45 Gy, 4.5 Gy/fraction) should be considered when the operation cannot be performed, or histological examination showed positive margins [30,31,32]. RT has been proven to effectively treat cutaneous adnexal tumors with matrical differentiation, with prescribed doses ranging from 25 to 60 Gy. Additionally, radiotherapy and computed tomography-guided interstitial high-dose-rate brachytherapy can be considered an integral aspect in the management of PC, with a positive margin or when excision cannot be performed, as primary or adjuvant treatment, in contrast to chemotherapy, which does not improve patient outcome (Figure 4) [29] (Table 2). The death related to PC was reported in ~7% of patients [25].

#### 2.1.2. Proliferating Trichilemmal Tumor

Proliferating trichilemmal tumor (PTT), which Wilson-Jones firstly described in 1966, is presumed to arise from trichilemmal cysts and may progress to a malignant PTT [33]. This process may be related to the loss of p53 tumor suppressor gene activity [11]. Instead of *TP53* mutation, the *PIK3CA* (Phosphatidylinositol-4,5-Bisphosphate 3-Kinase Catalytic Subunit Alpha) may play a role in pathogenesis (Table 1) [12].

This neoplasm most commonly occurs in women aged >40 years. In one meta-analysis, 185 cases were included and showed a local recurrence rate of 3.7%. Additionally, only about 60 cases of malignant tumors have been reported in the literature to date [34].

More than 90% of cases are located on the scalp. PTT usually appears as a solitary, slow-growing, exophytic lesion, 2–25 cm in size, before a rapid increase in the lesion’s size, which can be a sign of malignancy [35]. Proliferating trichilemmal tumors are rare lesions whose histologic hallmark is the presence of trichilemmal keratinization (Figure 3(B1–B3)). The keratin can form the globules, which can look like the keratin pearls in squamous cell carcinoma. Immunohistochemical markers, such as Ki-67, P53, AE13, AE14, and CD34, may help differentiate PTT with squamous cell carcinoma (SCC) [35,36]. It should be distinguished mainly from other neoplasms, which may be located in this region, and alopecia neoplastica (a rare form of skin metastasis in which internal malignancies spread to the scalp) [37]. Invasion of the surrounding tissue may be present. A desmoplastic stromal response is often seen. The number of mitoses varies widely, and the increased mitosis ratio may be related to the chance of malignancy [38]. Ye et al. proposed a distinction between benign (G1), locally aggressive (G2), and malignant variants (G3) based on tumor silhouette, degree of nuclear atypia, mitotic activity, necrosis, and perineurial or angiolymphatic invasion. There was a significant difference between these groups in recurrence and metastasis rate (G1 = 0%, G2 = 15%, G3 = 50%, *p* < 0.05). A basal cell carcinoma, sebaceous carcinoma, squamous cell carcinoma, angiosarcoma, clear cell hidradenocarcinoma, and other appendageal neoplasm have to be taken under consideration in the differential diagnosis of PTT [39].

An accepted treatment for benign PTT is simple local excision. If the histologic diagnosis of malignant type is made, more aggressive therapeutic measures such as nodal dissection, radiotherapy, or chemotherapy should be considered in addition to WLE or MMS, which provide superior margin evaluation that is extremely important in tumors with infiltrative borders [40]. If the histopathological diagnosis is a low-grade malignant tumor, the excision margin should be 1–2 cm due to its poorly understood biological behavior [12,39]. Cisplatin, 5-fluorouracil, adriamycin, and vindesine were used to treat this neoplasm with minimal success, especially as palliative treatment [41]. Lesions harboring an activating PI3K mutation seem to be sensitive to PI3K inhibitor (alpelisib). Gallant et al. reported a partial response for this drug in the patient with progression after surgery and adjuvant radiochemotherapy (weekly carboplatin plus paclitaxel). After 6 months, the patient died for widely metastatic MPTT, which was revealed during autopsy [12]. Adjuvant radiotherapy is widely used in case of metastases or local recurrence besides re-excision. Dubhashi et al. administered 42 Gy in 21 sessions throughout one month due to lymph node involvement [42]. Not all patients are good surgical candidates; neoadjuvant therapy can be used with complete response in some cases. Sutherland et al. reported the case of 93-year-old men who received 45 Gy over 3 weeks with a complete response and no evidence of primary tumors after 6 months (Table 2) [43]. Frequent clinical follow-up and lymph node examination are advised in the patient after wide excision of the malignant proliferating trichilemmal tumor due to late recurrences after diagnosis and treatment, which can occur from within 6 months to more than 10 years.

#### 2.1.3. Trichoblastic Carcinoma/Carcinosarcoma

Trichoblastic carcinoma/carcinosarcoma is a malignant and biphasic tumor made of two cell populations, epithelial and mesenchymal, differentiating toward the hair bulb region [44]. In some cases, the mutations in *TP53* and *CDKN2A* (Cyclin Dependent Kinase Inhibitor 2A) can be present (Table 1) [45].

Twelve cases have been reported in the literature since 2000 when it was described by Regauer et al. [46]. The trichoblastic carcinoma/carcinosarcoma most commonly occurs in men over 80 years old (age range: 34–92 years) [45].

The neoplasm size ranged from 0.9 cm to 20 cm [47]. The most frequent tumor sites are the head and the neck, followed by the shoulder and back. The cytologic atypia and conspicuous mitotic activity are present in both cells’ phases, which build the tumor. The primary differential diagnosis of a trichoblastic carcinosarcoma is sarcomatoid basal cell carcinoma (basal cell carcinosarcoma), which is diagnosed primarily in 94% of cases [48]. The epithelial component stains positive for keratins on the microscopic image, including CKAE1/3 (cytokeratin AE1/3) and CK5/6 (cytokeratin 5/6), and p63. The sarcomatous component is positive for vimentin, CD10 (cluster of differentiation 10), and p53. Both components are negative for EMA (epithelial membrane antigen), CD34 (cluster of differentiation 34), Mart-1 (melanoma antigen recognized by T cells 1), SOX10, actin, desmin, and CK20 (cytokeratin 20) [45,49].

According to the limited data, complete surgical excision with at least 1 cm margin follow-up and chest imaging consideration, particularly if immunocompromised, would be prudent. Interestingly, up to 57% of the patients required revision surgery due to positive margins [50]. Most cases have been recurrence-free. One patient suffered from chronic lymphocytic leukemia and succumbed after 21 months due to metastatic trichoblastic carcinoma. In that case, the patients received three cycles of chemotherapy (pharmorubicin-cisplatin), which caused massive tumor necrosis, followed by 3 months of radiotherapy (total 50 Gy) for another axillary recurrence [46]. Lepesant et al. administered vismodegib with remarkable response in metastatic trichoblastic carcinoma [51]. In another case, sunitinib was used with partial remission, and the disease stabilized after ten months (Table 2) [52].

#### 2.1.4. Tricholemmal Carcinoma

Tricholemmal carcinoma (TC) is an adnexal neoplasm, considered as the malignant form of trichilemmoma. It originates from the outer root sheath epithelium. Several risk factors have been described, including UV (ultraviolet) and ionizing radiation, preexisting scars, xeroderma pigmentosa, Cowden syndrome, or solid organs transplantation. Molecular basis includes *TP53* described in patients who had an aggressive clinical course and other mutations characteristic for other skin cancers, such as *TOP1* (DNA topoisomerase I), *NF1* (neurofibromin 1), or *NRAS* (Table 1) [11,14].

It is a rare condition; the largest group of reported cases subsumed into a publication is 103 patients included in a literature review [53]. The lesions’ location includes the face or scalp, with a slight majority of the cases reported in men. The predilection for a race is not clarified. TC usually affects elderly patients; the median age is approximately 72 years.

TC is observed as exophytic or polypoid mass. However, ulceration, telangiectasia, scales, or folded borders resembling basal cell carcinoma (BCC), (SCC), keratoacanthoma, or proliferating pilar cyst may be present as well. The average size of a malignant lesion is 2 cm at the diagnosis time, although the tumor can extend up to 25 cm [54]. Metastatic disease is remarkably rarely reported. Only a few cases in the literature, including a patient with immunosuppression due to kidney transplantation, and metastases are usually found in lymph nodes [54,55].

The histologic pattern of TC might resemble tricholemmoma. The lesion presents with lobular growth; peripheral palisade of cuboidal or columnar cells surrounds large and polygonal central cells, containing periodic acid-Schiff–positive, glycogen-rich cytoplasm. Cellular atypia and a high mitotic index are commonly reported (Figure 3(A1–A3)). Trichilemmal keratinization may be seen; the perineural invasion might be found as well. Immunohistochemical stainings are usually weak for EMA and CEA, though the expression of CK1, 10, 14, 17, and NGFR/p75 (nerve growth factor receptor) is present and suggests follicular infundibulum differentiation. However, some studies questioned using CK17 due to staining of the hair follicle’s inner root sheath from the infundibulum to the stem and the hair follicle companion cell layer [56]. TC loses CK15 and 16, contrary to tricholemmoma, which might correlate with malignant transformation of tricholemmoma to TC. Tricholemmomas show upregulation of CD34 expression without BerEP4 expression, which may be distinguishing between this tumor and basal cell carcinoma [57,58]. Lectin UEA-1 (Ulex europaeus agglutinin 1) is strongly positive in outer root sheath cells and TC but is not observed in SCC, BCC, actinic keratosis, or porocarcinoma.

Some dermatopathologists consider clear cell SCC (CCSCC) of the skin, a variant of SCC without trichilemmal differentiation, as equivalent entities. Even though glycogen-rich cytoplasm may be present in both these lesions, immunohistochemical markers help distinguish between them. Nonetheless, it is claimed that making that distinction is not worth additional efforts due to not clear difference in behavior or therapeutic approach [56].

Surgical excision is used in the therapy of this malignancy, preserving at least 1 cm surgical margins [59]. In some cases, MMS is performed, including recurrent local disease. Surgical treatment is usually sufficient—in most cases, there are no recurrences or metastases in follow-up. Jo et al. reported a case in which imiquimod 5% cream (used three times a week for 8 weeks) was used to treat TC when patients refused surgery without recurrence after 16 months [60]. Radiotherapy is considered in cases of unresectable lesions [61]. Adjuvant radiotherapy is also reported; Topkan et al. administered radiotherapy (60 Gy, 2 Gy per fraction) to a surgical bed with a 1 cm margin in a patient who underwent incomplete excision. No local, regional, or distant recurrences were found at the 49-month follow-up [62]. In another case, repeated adjuvant radiotherapy was unsuccessful in disease control [63]. Metastatic disease treatment includes chemotherapy and radiotherapy [64]. There is no established chemotherapy regimen reported in the medical literature. Cisplatin and cyclophosphamide combination chemotherapy was used with partial response [64]. Additionally, 5-fluorouracil combined with cisplatin was used without significant tumor size reduction (Table 2) [63].

In one analysis, the authors showed that the overall 5-year survival rate of the patients was 89.2%, with the presence of lymph node metastasis and status of surgery margin as key prognostic factors [65]. There are few critical prognostic factors for cutaneous carcinoma, including tumor size (>3 cm) and thickness, which seems to be consistent with researchers’ observation that neoplasms fulfilling these assumptions may pose a higher thread of metastatic or recurrent disease, so additional treatment, such as postoperative radiotherapy, should be considered [54].

### 2.2. Bening Tumors with Follicular Differentiation

#### 2.2.1. Trichoblastoma

Trichoblastomas can occur as a sporadic or syndromic lesions. Syndromic cases (occurring in the settings of multiple familial trichoepitheliomas and Brooke-Spiegler syndrome) are associated with germline mutations in *CYLD* [4,66]. No specific mutations are connected with the development of sporadic cases. In a minority of these, mutations in *CTNNB1*, *HRAS,* and *PTCH1* (Patched 1) have been found (Table 1) [10,15,16].

Trichoblastomas are most common during the 4th to 6th decade of life, with no significant sex bias [67].

**Figure 5:**
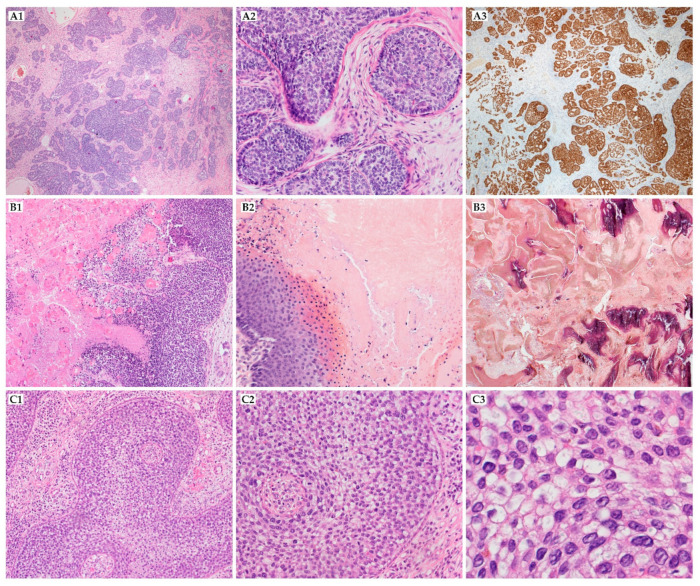
Benign tumors with follicular differentiation. (**A1**–**A3**) trichoblastoma: tumor composed of basaloid cells and specific stromal component resembling follicular mesenchyme [(**A1**) (20×, HE), (**A2**) (200×, HE)], the epithelial cells are strongly positive for BerEP4, it may be helpful in differential diagnosis with microcystic adnexal carcinoma which is infiltrative and negative for BerEP4 [(**A3**) (20×, BerEP4)]; (**B1**–**B3**) pilomatricoma, well-circumscribed lesion [(**B1**) (40×, HE)] with basophilic tumor with prominent cornification and numerous shadow cells [(**B2**) (100×, HE)], in older lesions calcifications and ossifications may be numerous [(**B3**) (20×, HE)]; (**C1**–**C3**) trichilemmoma: trichilemmoma, clear cell differentiation and prominent basement membrane, [(**C1**) (40×, HE), (**C2**) (200×, HE), (**C3**) (400×, HE)].

The typical clinical appearance of trichoblastoma is a skin-colored, slow-growing papule or nodule (0.5–3 cm in size), usually located on the face, neck, or scalp. It is crucial to distinguish trichoblastomas from BCCs. The lesions are limited to the dermis, in some cases involving the subcutis. The WHO classification divides the trichoblastomas into few variants: large nodular, small nodular, adamantinoid (lymphadenoma), retiform, racemiform, cribriform, and columnar trichoblastoma [1]. On a microscopic view, the neoplasms consist of homogenous basaloid cells and a stromal component (similar to follicular mesenchyme) (Figure 5(A1–A3)). The stroma is less abundant in adamantanoid, large nodular, and columnar trichoblastoma. The epithelial or follicular component can aggregate as small or large (with melanin deposits) nodules, which are the small and large nodular variants’ features. Adamantoid trichoblastomas are composed of epithelial nodules surrounded by palisaded basaloid cells and centrally located pale larger cells. The lymphocyte infiltration can be present. Columnar trichoblastoma consists of short cords, thin columns, and small nests composed of basaloid cells. In immunohistochemical staining, the epithelial component is positive for BerEP4 [1,68]. Trichoblastomas are CD10 and metalloendopeptidase positive [69]. The presence of CK20-positive Merkel cells in the basal layer is characteristic for trichoblastoma, but there are also cases without them [70]. There is evidence that none of the trichoblastomas expressed androgen receptors, whereas 78% of the BCCs expressed the receptors [71].

It is crucial to distinguish between BCCs and trichoblastomas. It can be difficult, and there exists a slight risk for malignant transformation; surgical excision is usually performed for trichoblastomas (Table 2). There is no recommendation for follow-up [67].

#### 2.2.2. Pilomatricoma

The exact intracellular signaling disorders are not known. The molecular study uncovers that the Wnt pathway can play a key crucial role in pilomatricoma (PM) development [17]. It takes part in regulating the β-catenin level in cells, which controls gene expression [18]. Hence, plenty of mutations can dysregulate the cell differentiation and promote carcinogenesis, e.g., exon three β-catenin activating mutations were found in 75% of cases of pilomatricoma [19]. Moreover, the mutation in APC-pathway can take part in pilomatrixoma development (Table 1) [6,20]. It was reported that this tumor could be the manifestation of Gardner syndrome [6,7]. Additionally, in some genetic syndromes, the pilomatrcomas can be present, especially as a part of myotonic dystrophy, Turner syndrome, Rubinstein–Taybi syndrome, Goldenhar syndrome, Kabuki syndrome, Sotos syndrome, trisomy 9, and DICER1 syndrome [1].

Pilomatrixoma is a benign tumor derived from the hair matrix. The mean age at excision was 16 years and 7 months with a range of 5 months to 97 years. It appears slightly more often in females [72,73]. Around 1% of all benign skin lesions are diagnosed as pilomatricoma [1].

The exact intracellular signaling disorders are not known. The molecular study uncovers that the Wnt pathway can play a crucial role in pilomatricoma (PM) development [17]. It takes part in regulating the β-catenin level in cells, which controls gene expression [18]. Hence, plenty of it mutations can dysregulate the cell differentiation and promote carcinogenesis, e.g., exon three β-catenin activating mutations were found in 75% of cases of pilomatricoma [19]. Additionally, the mutation in APC-pathway can take part in pilomatrixoma development (Table 1) [6,20]. It was reported that this tumor could be the manifestation of Gardner syndrome [6,7]. Additionally, in some genetic syndromes, the pilomatrcomas can be present, especially as a part of myotonic dystrophy, Turner syndrome, Rubinstein–Taybi syndrome, Goldenhar syndrome, Kabuki syndrome, Sotos syndrome, trisomy 9, and DICER1 syndrome [1]. Pilomatrixomas are reported to present as well-circumscribed bluish dermal swellings, sometimes present ulceration [25]. Typically, it is a slow-growing tumor, and sometimes rapid expansion with hemorrhage has been described [74]. In a recent review, the authors defined that commonest locations were the head and neck region (64%), the upper extremities (22%), the trunk (8%), and the lower extremities (5%), and the other part of the body (1%). In 72% of cases, the tumor was localized on the right side. Tumor diameters ranged from 0.5 to 20 cm (mean 3.8 cm, median 2.5 cm).

Pilomatricomas consist of one or several nodules located in the dermis; in some cases, hypodermis infiltration is observed. The peripheral cells are small, homogenous, and basophilic with high mitotic activity. The centrally located cells are keratinized, eosinophilic with distinct borders. These cells are named as ghost or shadow cells (Figure 5(B1–B3)). In some cases, the cystic variant can be diagnosed, especially in syndromic cases (mainly in Gardner syndrome). Additionally, the granulocytes infiltration and fibrosis can be present [1]. The identification of ghost and basaloid cells is the basis of a diagnosis of a pilomatrixoma [72].

The definitive treatment is complete excision with clear margins. The spontaneous regression of pilomatricoma has never been observed. Pilomatrixomas commonly occur on cosmetically sensitive areas, and incomplete resection almost always results in recurrence. In a recent review, authors support that complete resection is characterized by low recurrence rates (1.4%) [72]. Adjuvant and neoadjuvant therapy are not needed (Table 2) [73].

#### 2.2.3. Trichilemmoma

Trichilemmoma is a benign follicular tumor with differentiation toward the outer root sheath of the pilosebaceous follicular epithelium, developing slowly over months to years [57,75]. Nature and pathogenesis have been a matter of debate for a long time; however, activating HRAS mutations, not only the same which occur in naevus sebaceous’ genotype, are carried; thus, molecular evidence indicates that most trichilemmomas are authentic neoplasms [22]. Additionally, complete loss of PTEN staining occurs in >80% of trichilemmomas in patients with Cowden syndrome and only 3% of sporadic ones (Table 1). Multiple risk factors have been identified, including UV and ionizing radiation, previous trauma or scar, genetic disorders (xeroderma pigmentosum and Cowden disease), and immunosuppression associated with solid organ transplantation. Tumors may also grow secondary to naevus sebaceous or be histologically related to BCC [76,77,78]. It has been hypothesized that human papillomavirus (HPV) might have a pathogenetic role. However, variable researches showed no linkage between the histogenesis of trichilemmoma and HPV [79,80].

Lesions are typically encountered in middle-aged adults, with a predilection for males [81]. It primarily affects the face; tumors also occur on the scalp, neck, or chest [75].

Trichilemmoma usually presents with a nondescript wart-like or smooth dome-shaped appearance, <1 cm in diameter. The surface is typically smooth or crusted; it may exhibit pearly edges. Lesions may be multiple [81].

In histological examination, a squamo-proliferative lesion consists of pale eosinophilic or clear cells with a peripheral palisade of cuboidal or columnar cells positioned on basement membrane similar to the hair outer root sheath area below the level of the follicular isthmus; the growth is lobular (Figure 5(C1–C3)). CK1, CK10, CK17, CD34, and D2-40 expression patterns, characteristic for the outer root sheath of normal hair follicles, are typically observed in trichilemmonas [58]. Other immunohistochemical stains, such as p16, are also diagnostic [75,82,83,84]. Desmoplastic trichilemmoma, a histological variant, presents with a pseudocarcinomatous pattern, including a prominent fibrotic or desmoplastic stroma, but without nuclear atypia or mitotic activity [78].

Treatment of trichilemmoma is usually surgical excision; however, the recurrence rate after surgery is unknown (Table 2) [78].

#### 2.2.4. Trichofolliculoma

Trichofolliculoma is a rare hamartomatous lesion with follicular differentiation. The pathogenesis has not known. Some authors suggest that signaling pathways involving BMP and PYG02 are associated with the experimental development of trichofolliculoma-like proliferations (Table 1) [23].

Trichofolliculoma has a slight male predominance and a predilection for middle-aged adults [85]. The congenital cases are sporadic, but they were reported in the literature [86,87].

The vast majority of trichofolliculomas are located on the face and the neck. The hair follicle grows to the skin surface by an opening that is seen macroscopically. Several smaller follicles can radiate to the surrounding from this primary follicle [1]. The lesions sometimes produce fully developed hair, resulting in a clinical appearance of multiple hairs growing from a papule [88]. From a microscopic view, it is a primary infundibular cystic structure in connection with the overlying epidermis with follicle buds [89]. Trichofolliculoma expresses CK17 intensely, as well as PHLDA1 (Pleckstrin homology like domain family A member 1) and BerEP4 [85].

There are no recommendations due to insufficient data. In most cases, the wide local excision was performed with no recurrences (Table 2).

#### 2.2.5. Pilar Sheath Acanthoma

Pilar sheath acanthoma is a benign follicular neoplasm [90]. Some authors suggest that this tumor is a benign follicular hamartoma. There are no known genetic abnormalities described in patients with this neoplasm [91].

Pilar sheath acanthoma is rare. It typically affects middle-aged and elderly people (age range: 30–76 years), with no sex predilection [91].

The most common location for pilar sheath acanthoma is the face and the neck. The tumor is an endophytic dermal lesion [92]. It is asymptomatic, small flesh-colored nodule (diameter 0.3–0.5 cm). The lesion has two components—a superficial infundibular component and a deeper component made up of solid epithelial lobules, which show keratinization similar to that of the outer root sheath at the level of the isthmus of the hair follicle [2,91].

The observation is recommended, but due to cosmetic reasons, the lesion is usually removed and, after simple excision, does not require further treatment (Table 2).

#### 2.2.6. Tumor of the Follicular Infundibulum

Tumor of the follicular infundibulum (TFI) is an uncommon benign skin lesion. Few theories which try to explain the development of this lesion consist. Some authors suggest that TFI represents a histological reaction pattern for the other neoplasm [93]. Another theory is that it represents a form of BCC [94]. However, this explanation would not explain the association of TFI with neoplasms other than. The latest analysis showed that this lesion can be an epidermal pattern caused by scarring [95]. Still, it is not conclusive which theory is correct. There are no specific mutations connected with the development of TFI.

TFI is a rare tumor, diagnosed with a relative frequency of 3–20 cases per 100,000 skin biopsies. It affects mainly older adults (mean age 67 years old) with no marked sex predominance [93,94,95].

This neoplasm usually presents as a solitary keratotic papule or plaque on the face or scalp. The tumoral cells exhibit a reticulated growth pattern and at the periphery may be arrayed in palisade. The tubular structures in TFI stain positive for calretinin; the neoplastic cells contain glycogen, suggesting differentiation toward the outer root sheath [93,94,95].

Both the solitary and multiple cases are benign; however, documented transformation to basal cell carcinoma has been found, and subsequent follow-ups are needed [93,96]. In the majority of cases, the excision was performed due to esthetical reasons. The local treatment (topical keratolytic, retinoid acid or steroids, cryotherapy) can be considered in some cases (Table 2).

#### 2.2.7. Melanocytic Matricoma

Some authors have proposed that the presence of melanocytes and matrical cells in a lesion should be expected due to the interaction between these cells during hair cycling [97]. Solar radiation may also play a role in the development of melanocytic matricoma [98].

Approximately 20 cases have been reported in the literature. This tumor typically affects elderly individuals with a male predilection (male to female ratio, 4.5:1) [98].

Melanocytic matricoma manifests as a small, pigmented, purple, or brownish-black papule or nodule, predominantly on sun-damaged skin, which may be ulcerated or crusted, and painful. The tumor is built from basophilic germinative hair matrix cells and melanin deposits with pigmented melanocytes. The matrical cells express PHLDA1 and β-catenin (in the nucleus), but not BerEP4 [98]. Intratumoral melanocytes stain positive for S100, HMB45, and melan-A [98,99].

Melanocytic matricoma is benign. Some cases with prominent melanocytic and matrical atypia have sometimes been reported as malignant melanocytic matricoma, but their clinical behavior has not been well characterized [100]. In these cases, the complete excision with subsequent follow-up should be considered (Table 2).

#### 2.2.8. Spindle Cell-Predominant Trichodiscoma

Spindle cell-predominant trichodiscoma (SCPT) is a hamartomatous tumor of the follicular unit [101]. The genetic profile is unknown.

SCPT affects mainly people after the third decade of life (mean age: 58 years old). There is no sex predominance [102].

This neoplasm occurs predominantly on the nose [101]. SCPT typically presents as a solitary, protuberant, usually dome-shaped, skin-colored, and smooth-surfaced papule. The neoplasm is a symmetrical tumor composed of hyperplastic sebaceous lobules and central mucinous stroma with fibrocytes proliferation, which show elongated, slightly wavy nuclei and delicate tapering cytoplasm. SCPT stains positive for CD34 and negative for S100 protein [101,102].

Local excision without perioperative therapy provides an excellent local control rate (Table 2) [1].

## 3. Conclusions

Macroscopic presentation of each of presented tumor is unspecific, what means that histological examination is required. Differential diagnosis includes BCC, SCC, sebaceous carcinoma, angiosarcoma, clear cell hidradenocarcinoma, other lesions located on the skin, especially other appendageal neoplasms and alopecia neoplastica [1,37]. Dermatoscopy is not recommended for diagnosis because of the unspecific manifestation of each tumor (most lesions have abnormal vascular patterns, areas of calcification, or keratin masses) [103]. The excisional or punch biopsy is a gold standard because superficial shave biopsies may miss relevant structures [1]. The similarities in microscopic views between tumors make that diagnosis should be performed in a high-reference oncology center. The overlapping of benign and malignant lesions can make difficulties, and distinguish between them is essential for correct prognosis, administration of appropriate treatment, and follow-up. The essential information on diagnosis is included in the section about each neoplasm.

In most cases, the adnexal tumors derived from follicular tissue are benign lesions. There is no treatment algorithm because some of these tumors are extremely rare. The wide excision is the treatment of choice. The local treatment (topical keratolytic, retinoid acid or steroids, cryotherapy) can be considered, but the impact on outcome is not confirmed, so it should not be recommended as a standard. In some cases, especially in highly aggressive tumors, the adjuvant treatment was applied (Table 2). More analyses are needed to determine the proper management of the patient.

**Table 2:** The multidisciplinary treatment cases reported for appendageal tumors.

Cancer Type	Surgery	Chemotherapy (Agents)	Reference	Radiotherapy	Reference
Pilomatrical carcinoma	R	NR	[25,29,72,104]	A or NA: 45 Gy, 4.5 Gy/fraction; A after LND	[25,29,30,31,32,72,104,105]
Proliferating trichilemmal tumor	R	MD: cisplatin, doxorubicin, vinca alkaloids, 5-fluorouracil PI3K inhibitor	[12,41,43,106]	A, MD	[12,39,42,43,61,106]
Trichoblastic carcinoma/carcinosarcoma	R	pharmorubicin-cisplatin, vismodegib, sunitinib	[45,46,51,52,107]	LD	[45,46,107,108,109]
Tricholemmal carcinoma	R	LD	[54,59,63,64,65,110]	LD	[54,62,63]
Trichoblastoma	R	ND		ND	
Pilomatrixoma	R	ND		ND	
Trichilemmoma	R	ND		ND	
Trichofolliculoma	R	ND		ND	
Pilar sheath acanthoma	R	ND		ND	
Tumor of the follicular infundibulum	R	ND		ND	
Melanocytic matricoma	R	ND		ND	
Spindle cell-predominant trichodiscoma	R	ND		ND	

R—recommended; NR—not recommended; ND—no data; NA—neoadjuvant therapy; A—adjuvant therapy; LR—local recurrence; NM—nodal metastasis; MD—metastatic disease; LD—limited data.

## Figures and Tables

**Table 1:** Genetic abnormalities reported in appendageal tumors.

Cancer Type	Genetic Abnormalities			Reference
	Mutation	Expression Downregulated	Expression Upregulated	
Pilomatrical carcinoma	*CTNNB1*, *CDX2, LEF-1*		*CDX2, LEF-1*	[9,10]
Proliferating trichilemmal tumor	*TP53, PI3KCA*	*TP53*	*PIK3*	[11,12,13]
Trichoblastic carcinoma/carcinosarcoma	*TP53, CDKN2A*			
Trichilemmal carcinoma	*TP53, NF1, TOP1, PTEN*	*PTEN, TP53*	*TOP1*	[14]
Trichoblastoma	*CYLD, CTNNB1, HRAS, PTCH1*		*HRAS*	[10,15,16]
Pilomatrixoma	*CTNNB1, BCL2, APC, CTNNB1*			[6,17,18,19,20,21]
Trichilemmoma	*HRAS, PTEN*		*HRAS*	[22]
Trichofolliculoma	*BMP, PYGO2* (only experimental models)			[23]
Pilar sheath acanthoma	ND			
Tumor of the follicular infundibulum	ND			
Melanocytic matricoma	ND			
Spindle cell-predominant trichodiscoma	ND			

## Data Availability

Raw pathology figures data was generated at Department of Pathology and Laboratory Diagnostics, Maria Sklodowska-Curie National Research Institute of Oncology and are available from the authors on request.

## References

[1] Elder D.E., Massi D., Scolyer R.A., Willemze R. (2018). WHO Classification of Skin Tumours.

[2] Tellechea O., Cardoso J.C., Reis J.P., Ramos L., Gameiro A.R., Coutinho I., Baptista A.P. (2015). Benign follicular tumors. Bras. Derm..

[3] Ashinoff R., Jacobson M., Belsito D.V. (1993). Rombo syndrome: A second case report and review. J. Am. Acad. Dermatol..

[4] Kazakov D.V. (2016). Brooke-Spiegler Syndrome and Phenotypic Variants: An Update. Head Neck Pathol..

[5] Farooq A., Walker L.J., Bowling J., Audisio R.A. (2010). Cowden syndrome. Cancer Treat. Rev..

[6] Stevenson P., Rodins K., Susman R. (2018). The association between multiple pilomatrixomas and APC gene mutations. Australas J. Derm..

[7] Trufant J., Kurz W., Frankel A., Muthusamy V., McKinnon W., Greenblatt M., Lazar A., Cook D., Bosenberg M. (2012). Familial multiple pilomatrixomas as a presentation of attenuated adenomatosis polyposis coli. J. Cutan. Pathol..

[8] Rohwedder A., Keminer O., Hendricks C., Schaller J. (1997). Detection of HPV DNA in trichilemmomas by polymerase chain reaction. J. Med. Virol..

[9] Tumminello K., Hosler G.A. (2018). CDX2 and LEF-1 expression in pilomatrical tumors and their utility in the diagnosis of pilomatrical carcinoma. J. Cutan. Pathol..

[10] Kazakov D.V., Sima R., Vanecek T., Kutzner H., Palmedo G., Kacerovska D., Grossmann P., Michal M. (2009). Mutations in exon 3 of the CTNNB1 gene (beta-catenin gene) in cutaneous adnexal tumors. Am. J. Derm..

[11] Takata M., Rehman I., Rees J.L. (1998). A trichilemmal carcinoma arising from a proliferating trichilemmal cyst: The loss of the wild-type p53 is a critical event in malignant transformation. Hum. Pathol..

[12] Gallant J.N., Sewell A., Almodovar K., Wang Q., Dahlman K.B., Abramson R.G., Kapp M.E., Brown B.T., Boyd K.L., Gilbert J. (2019). Genomic landscape of a metastatic malignant proliferating tricholemmal tumor and its response to PI3K inhibition. NPJ Precis. Oncol..

[13] Nakai N., Takenaka H., Hamada S., Kishimoto S. (2008). Identical p53 gene mutation in malignant proliferating trichilemmal tumour of the scalp and small cell carcinoma of the common bile duct: The necessity for therapeutic caution?. Br. J. Derm..

[14] Ha J.H., Lee C., Lee K.S., Pak C.S., Sun C.H., Koh Y., Chang H. (2020). The molecular pathogenesis of Trichilemmal carcinoma. BMC Cancer.

[15] Shen A.S., Peterhof E., Kind P., Rutten A., Zelger B., Landthaler M., Berneburg M., Hafner C., Groesser L. (2015). Activating mutations in the RAS/mitogen-activated protein kinase signaling pathway in sporadic trichoblastoma and syringocystadenoma papilliferum. Hum. Pathol..

[16] Hafner C., Schmiemann V., Ruetten A., Coras B., Landthaler M., Reifenberger J., Vogt T. (2007). PTCH mutations are not mainly involved in the pathogenesis of sporadic trichoblastomas. Hum. Pathol..

[17] Lazar A.J., Calonje E., Grayson W., Dei Tos A.P., Mihm M.C., Redston M., McKee P.H. (2005). Pilomatrix carcinomas contain mutations in CTNNB1, the gene encoding beta-catenin. J. Cutan. Pathol..

[18] Duchartre Y., Kim Y.M., Kahn M. (2016). The Wnt signaling pathway in cancer. Crit. Rev. Oncol. Hematol..

[19] Hassanein A.M., Glanz S.M. (2004). Beta-catenin expression in benign and malignant pilomatrix neoplasms. Br. J. Derm..

[20] Urabe K., Xia J., Masuda T., Moroi Y., Furue M., Matsumoto T. (2004). Pilomatricoma-like changes in the epidermoid cysts of Gardner syndrome with an APC gene mutation. J. Derm..

[21] Agoston A.T., Liang C.W., Richkind K.E., Fletcher J.A., Vargas S.O. (2010). Trisomy 18 is a consistent cytogenetic feature in pilomatricoma. Mod. Pathol..

[22] Tsai J.H., Huang W.C., Jhuang J.Y., Jeng Y.M., Cheng M.L., Chiu H.Y., Kuo K.T., Liau J.Y. (2014). Frequent activating HRAS mutations in trichilemmoma. Br. J. Derm..

[23] Sun P., Watanabe K., Fallahi M., Lee B., Afetian M.E., Rheaume C., Wu D., Horsley V., Dai X. (2014). Pygo2 regulates beta-catenin-induced activation of hair follicle stem/progenitor cells and skin hyperplasia. Proc. Natl. Acad. Sci. USA.

[24] Bremnes R.M., Kvamme J.M., Stalsberg H., Jacobsen E.A. (1999). Pilomatrix carcinoma with multiple metastases: Report of a case and review of the literature. Eur. J. Cancer.

[25] Herrmann J.L., Allan A., Trapp K.M., Morgan M.B. (2014). Pilomatrix carcinoma: 13 new cases and review of the literature with emphasis on predictors of metastasis. J. Am. Acad. Derm..

[26] Nogal P., Bartkowiak E., Iwanik K., Wierzbicka M. (2020). Common sense and tumor treatment. A case of pilomatrical carcinoma in a 21-year-old patient with surprisingly rapid tumor progression. Oral Oncol..

[27] Sable D., Snow S.N. (2004). Pilomatrix carcinoma of the back treated by mohs micrographic surgery. Derm. Surg..

[28] Papadakis M., de Bree E., Floros N., Giannikaki E., Xekalou A., Manios A. (2017). Pilomatrix carcinoma: More malignant biological behavior than was considered in the past. Mol. Clin. Oncol..

[29] Tselis N., Heyd R., Vogt H.G., Zamboglou N. (2006). Pilomatrix carcinoma with lymph node and pulmonary metastases. Strahlenther Onkol..

[30] Sau P., Lupton G.P., Graham J.H. (1993). Pilomatrix carcinoma. Cancer.

[31] Gould E., Kurzon R., Kowalczyk A.P., Saldana M. (1984). Pilomatrix carcinoma with pulmonary metastasis. Report of a case. Cancer.

[32] Waqas O., Faisal M., Haider I., Amjad A., Jamshed A., Hussain R. (2017). Retrospective study of rare cutaneous malignant adnexal tumors of the head and neck in a tertiary care cancer hospital: A case series. J. Med. Case Rep..

[33] Jones E.W. (1966). Proliferating Epidermoid Cysts. Arch. Dermatol..

[34] ElBenaye J., Sinaa M., Elkhachine Y., Sakkah A., Jakar A., Elhaouri M. (2018). Proliferating trichilemmal cyst of the scalp: Nine cases and literature review. Otorhinolaryngol. Head Neck Surg..

[35] Satyaprakash A.K., Sheehan D.J., Sangueza O.P. (2007). Proliferating trichilemmal tumors: A review of the literature. Derm. Surg.

[36] Alici O., Keles M.K., Kurt A. (2015). A Rare Cutaneous Adnexal Tumor: Malignant Proliferating Trichilemmal Tumor. Case Rep. Med..

[37] Paolino G., Pampena R., Grassi S., Mercuri S.R., Cardone M., Corsetti P., Moliterni E., Muscianese M., Rossi A., Frascione P. (2019). Alopecia neoplastica as a sign of visceral malignancies: A systematic review. J. Eur. Acad. Derm. Venereol..

[38] Adegun O.K., Morley S., Kalavrezos N., Jay A. (2019). Proliferating trichilemmal tumour: Diagnostic challenge on core biopsy. BMJ Case Rep..

[39] Ye J., Nappi O., Swanson P.E., Patterson J.W., Wick M.R. (2004). Proliferating Pilar Tumors. Am. J. Clin. Pathol..

[40] Fieleke D.R., Goldstein G.D. (2015). Malignant proliferating trichilemmal tumor treated with Mohs surgery: Proposed protocol for diagnostic work-up and treatment. Derm. Surg..

[41] Hayashi I., Harada T., Muraoka M., Ishii M. (2004). Malignant proliferating trichilemmal tumour and CAV (cisplatin, adriamycin, vindesine) treatment. Br. J. Derm..

[42] Dubhashi S.P., Jadhav S.K., Parasnis A., Patil C.S. (2014). Recurrent malignant proliferating trichilemmal tumor with lymph node metastasis in a young woman. J. Postgrad. Med..

[43] Sutherland D., Roth K., Yu E. (2017). Malignant Proliferating Trichilemmal Tumor Treated with Radical Radiotherapy: A Case Report and Literature Review. Cureus.

[44] Tran T.A., Muller S., Chaudahri P.J., Carlson J.A. (2005). Cutaneous carcinosarcoma: Adnexal vs. epidermal types define high- and low-risk tumors. Results of a meta-analysis. J. Cutan. Pathol..

[45] Kim C., Brown A., Osipov V. (2021). Trichoblastic carcinosarcoma in a 34-year-old woman with histopathologic and molecular analysis, including re-demonstration of a CDKN2A p.(R58*) mutation. J. Cutan. Pathol..

[46] Regauer S., Beham-Schmid C., Okcu M., Hartner E., Mannweiler S. (2000). Trichoblastic carcinoma (“malignant trichoblastoma”) with lymphatic and hematogenous metastases. Mod. Pathol..

[47] Nair I.M., Gharpuray-Pandit D., Cardozo C. (2021). A case of trichoblastic carcinosarcoma with review of literature. Hum. Pathol. Case Rep..

[48] Yaacoub E., El Borgi J., Challita R., Sleiman Z., Ghanime G. (2020). Pinna high grade trichoblastic carcinoma, a report. Clin. Pr..

[49] Li J.J.X., Ng J.K.M., Choi P.C.L., Lee J.H.S., Yu M.Y. (2020). Trichoblastic Carcinosarcoma Arising From the Vagina: A Case Report With Comprehensive Immunophenotypic Analysis. Int. J. Surg. Pathol..

[50] Thomas M., Bruant-Rodier C., Bodin F., Cribier B., Huther M., Dissaux C. (2017). Why is it important to differentiate trichoblastic carcinomas (CT) from basal cell carcinomas (CBC). About 21 cases. Ann. Chir. Plast. Esthet..

[51] Lepesant P., Crinquette M., Alkeraye S., Mirabel X., Dziwniel V., Cribier B., Mortier L. (2015). Vismodegib induces significant clinical response in locally advanced trichoblastic carcinoma. Br. J. Derm..

[52] Battistella M., Mateus C., Lassau N., Chami L., Boukoucha M., Duvillard P., Cribier B., Robert C. (2010). Sunitinib efficacy in the treatment of metastatic skin adnexal carcinomas: Report of two patients with hidradenocarcinoma and trichoblastic carcinoma. J. Eur. Acad. Derm. Venereol..

[53] Hamman M.S., Brian Jiang S.I. (2014). Management of trichilemmal carcinoma: An update and comprehensive review of the literature. Derm. Surg..

[54] Feng Z., Zhu H.G., Wang L.Z., Zheng J.W., Chen W.T., Zhang Z., Dong W., Qu W., Wang Y.A. (2014). Tricholemmal carcinoma of the head and neck region: A report of 15 cases. Oncol. Lett..

[55] Hiramatsu K., Sasaki K., Matsuda M., Hashimoto M., Eguchi T., Tomikawa S., Fujii T., Watanabe G. (2015). A case of trichilemmal carcinoma with distant metastases in a kidney transplantation patient. Transpl. Proc..

[56] Dalton S.R., LeBoit P.E. (2008). Squamous cell carcinoma with clear cells: How often is there evidence of tricholemmal differentiation?. Am. J. Derm..

[57] Turnbull N., Ghumra W., Mudaliar V., Vella J., Sanders D.S.A., Taibjee S., Carr R. (2018). CD34 and BerEP4 Are Helpful to Distinguish Basaloid Tricholemmoma From Basal Cell Carcinoma. Am. J. Derm..

[58] Misago N., Toda S., Narisawa Y. (2012). Tricholemmoma and clear cell squamous cell carcinoma (associated with Bowen’s disease): Immunohistochemical profile in comparison to normal hair follicles. Am. J. Derm..

[59] Xu B., Wang T., Liao Z. (2018). Surgical Treatment of Trichilemmal Carcinoma. World J. Oncol..

[60] Jo J.H., Ko H.C., Jang H.S., Kim M.B., Oh C.K., Kwon K.S. (2005). Infiltrative trichilemmal carcinoma treated with 5% imiquimod cream. Derm. Surg..

[61] Parambeth H.K., Udhayam N., Agarwal S., Gupta S., Giridhar P., Rath G.K. (2019). A large helmet-shaped proliferating trichilemmal tumor of the scalp: Is definitive radiotherapy the treatment? A case report. J. Egypt Natl. Cancer Inst..

[62] Topkan E. (2016). Adjuvant radiotherapy in management of trichilemmal carcinoma of left nasal alae with positive surgical margins: A case report. Case Rep. Clin. Pathol..

[63] Roismann M., Freitas R.R., Ribeiro L.C., Montenegro M.F., Biasi L.J., Jung J.E. (2011). Trichilemmal carcinoma: Case report. Bras. Derm..

[64] Yi H.S., Sym S.J., Park J., Cho E.K., Ha S.Y., Shin D.B., Lee J.H. (2010). Recurrent and metastatic trichilemmal carcinoma of the skin over the thigh: A case report. Cancer Res. Treat..

[65] Zhuang S.M., Zhang G.H., Chen W.K., Chen S.W., Wang L.P., Li H., Song M. (2013). Survival study and clinicopathological evaluation of trichilemmal carcinoma. Mol. Clin. Oncol..

[66] Kazakov D.V., Vanecek T., Nemcova J., Kacerovska D., Spagnolo D.V., Mukensnabl P., Michal M. (2009). Spectrum of tumors with follicular differentiation in a patient with the clinical phenotype of multiple familial trichoepitheliomas: A clinicopathological and molecular biological study, including analysis of the CYLD and PTCH genes. Am. J. Derm..

[67] Pina A., Sauthier P., Rahimi K. (2015). Vulvar trichoblastoma: Case report and literature review. J. Low Genit. Tract Dis..

[68] Patel P., Nawrocki S., Hinther K., Khachemoune A. (2020). Trichoblastomas Mimicking Basal Cell Carcinoma: The Importance of Identification and Differentiation. Cureus.

[69] Cordoba A., Guerrero D., Larrinaga B., Iglesias M.E., Arrechea M.A., Yanguas J.I. (2009). Bcl-2 and CD10 expression in the differential diagnosis of trichoblastoma, basal cell carcinoma, and basal cell carcinoma with follicular differentiation. Int. J. Derm..

[70] Schulz T., Hartschuh W. (1997). Merkel cells are absent in basal cell carcinomas but frequently found in trichoblastomas. An immunohistochemical study. J. Cutan. Pathol..

[71] Izikson L., Bhan A., Zembowicz A. (2005). Androgen receptor expression helps to differentiate basal cell carcinoma from benign trichoblastic tumors. Am. J. Derm..

[72] Jones C.D., Ho W., Robertson B.F., Gunn E., Morley S. (2018). Pilomatrixoma: A Comprehensive Review of the Literature. Am. J. Derm..

[73] Schwarz Y., Pitaro J., Waissbluth S., Daniel S.J. (2016). Review of pediatric head and neck pilomatrixoma. Int. J. Pediatr. Otorhinolaryngol..

[74] Swerlick R.A., Cooper P.H., Mackel S.E. (1982). Rapid enlargement of pilomatricoma. J. Am. Acad. Dermatol..

[75] Zhong S., Wang L., Mei X.L. (2019). Desmoplastic trichilemmoma of the scalp: Case report and literature review of immunohistochemical staining features. J. Int. Med. Res..

[76] Hsu M.C., Liau J.Y., Hong J.L., Cheng Y., Liao Y.H., Chen J.S., Sheen Y.S., Hong J.B. (2016). Secondary neoplasms arising from nevus sebaceus: A retrospective study of 450 cases in Taiwan. J. Derm..

[77] Zaballos P., Serrano P., Flores G., Bañuls J., Thomas L., Llambrich A., Castro E., Lallas A., Argenziano G., Zalaudek I. (2015). Dermoscopy of tumours arising in naevus sebaceous: A morphological study of 58 cases. J. Eur. Acad. Derm. Venereol..

[78] Pihlblad M., Chelnis J., Schaefer D. (2014). Eyelid desmoplastic trichilemmoma: 2 case reports and review. Ophthalmic Plast. Reconstr. Surg..

[79] Stierman S., Chen S., Nuovo G., Thomas J. (2010). Detection of human papillomavirus infection in trichilemmomas and verrucae using in situ hybridization. J. Cutan. Pathol..

[80] Leonardi C.L., Zhu W.Y., Kinsey W.H., Penneys N.S. (1991). Trichilemmomas are not associated with human papillomavirus DNA. J. Cutan. Pathol..

[81] Maher E.E., Vidal C.I. (2015). Trichilemmoma. Cutis.

[82] Hilliard N.J., Wakefield D.N., Krahl D., Sellheyer K. (2009). p16 expression in conventional and desmoplastic trichilemmomas. Am. J. Derm..

[83] Tardío J.C. (2009). CD34-reactive tumors of the skin. An updated review of an ever-growing list of lesions. J. Cutan. Pathol..

[84] Kurokawa I., Nishijima S., Kusumoto K., Senzaki H., Shikata N., Tsubura A. (2003). Trichilemmoma: An immunohistochemical study of cytokeratins. Br. J. Derm..

[85] Romero-Perez D., Garcia-Bustinduy M., Cribier B. (2017). Clinicopathologic study of 90 cases of trichofolliculoma. J. Eur. Acad. Derm. Venereol..

[86] Al-Ghadeer H., Edward D.P. (2017). Congenital Sebaceous Trichofolliculoma of the Upper Eyelid. Ophthalmic Plast Reconstr. Surg.

[87] Ishii N., Kawaguchi H., Takahashi K., Nakajima H. (1992). A case of congenital trichofolliculoma. J. Derm..

[88] Misago N., Ansai S.I., Fukumoto T., Anan T., Kimura T., Nakao T. (2017). Chronological changes in trichofolliculoma: Folliculosebaceous cystic hamartoma is not a very-late-stage trichofolliculoma. J. Derm..

[89] Kapoor A.G., Vijitha V.S., Mittal R. (2019). Trichofolliculoma of the Eyelid Margin: A Case Report and Review of Literature. Ophthalmic Plast. Reconstr. Surg..

[90] Bhawan J. (1979). Pilar sheath acanthoma. A new benign follicular tumor. J. Cutan. Pathol..

[91] Jo-Velasco M., Corrales-Rodriguez A., Frances-Rodriguez L., Alegria-Landa V., Erana-Tomas I., Rutten A., Requena L. (2018). Plaque-Like Pilar Sheath Acanthoma: Histopathologic and Immunohistochemical Study of 3 Unusual Cases. Am. J. Derm..

[92] Kushner J.A., Thomas R.S., Young R.J. (2014). An unusual location of a pilar sheath acanthoma. Int. J. Trichology.

[93] Abbas O., Mahalingam M. (2009). Tumor of the follicular infundibulum: An epidermal reaction pattern?. Am. J. Derm..

[94] Weyers W., Horster S., Diaz-Cascajo C. (2009). Tumor of follicular infundibulum is Basal cell carcinoma. Am. J. Derm..

[95] Baquerizo Nole K.L., Lopez-Garcia D.R., Teague D.J., Al Sayyah A., Mansoori P., Salim Al Alshehri H., Sangueza O.P. (2015). Is Tumor of Follicular Infundibulum a Reaction to Dermal Scarring?. Am. J. Derm..

[96] Cheng A.C., Chang Y.L., Wu Y.Y., Hu S.L., Chuan M.T. (2004). Multiple tumors of the follicular infundibulum. Derm. Surg..

[97] Carlson J.A., Healy K., Slominski A., Mihm M.C. (1999). Melanocytic matricoma: A report of two cases of a new entity. Am. J. Derm..

[98] Zussman J., Sheth S., Ra S.H., Binder S.W. (2011). Melanocytic matricoma with melanocytic atypia: Report of a unique case and review of the literature. Am. J. Derm..

[99] Barrado-Solis N., Moles-Poveda P., Roca-Estelles M.J., Quecedo-Estebanez E., Gimeno-Carpio E. (2016). Melanocytic matricoma with melanocytic atypia: Report of a new case. J. Eur. Acad. Derm. Venereol..

[100] Ardakani N.M., Palmer D.L., Wood B.A. (2016). Malignant Melanocytic Matricoma: A Report of 2 Cases and Review of the Literature. Am. J. Derm..

[101] Michalova K., Kutzner H., Steiner P., Hadravsky L., Michal M., Michal M., Kazakov D.V. (2019). Spindle Cell Predominant Trichodiscoma or Spindle Cell Lipoma With Adnexal Induction? A Study of 25 Cases, Revealing a Subset of Cases With RB1 Heterozygous Deletion in the Spindle Cell Stroma. Am. J. Derm..

[102] Kutzner H., Requena L., Rutten A., Mentzel T. (2006). Spindle cell predominant trichodiscoma: A fibrofolliculoma/trichodiscoma variant considered formerly to be a neurofollicular hamartoma: A clinicopathological and immunohistochemical analysis of 17 cases. Am. J. Derm..

[103] Zaballos P., Gomez-Martin I., Martin J.M., Banuls J. (2018). Dermoscopy of Adnexal Tumors. Derm. Clin..

[104] Mikhaeel N.G., Spittle M.F. (2001). Malignant pilomatrixoma with multiple local recurrences and distant metastases: A case report and review of the literature. Clin. Oncol..

[105] Hardisson D., Linares M.D., Cuevas-Santos J., Contreras F. (2001). Pilomatrix carcinoma: A clinicopathologic study of six cases and review of the literature. Am. J. Derm..

[106] Lobo L., Amonkar A.D., Dontamsetty V.V. (2016). Malignant Proliferating Trichilemmal Tumour of the Scalp with Intra-Cranial Extension and Lung Metastasis-a Case Report. Indian J. Surg..

[107] Okhremchuk I., Nguyen A.T., Fouet B., Morand J.J. (2018). Trichoblastic Carcinosarcoma of the Skin: A Case Report and Literature Review. Am. J. Derm..

[108] Laffay L., Depaepe L., d’Hombres A., Balme B., Thomas L., De Bari B. (2012). Histological features and treatment approach of trichoblastic carcinomas: From a case report to a review of the literature. Tumori.

[109] Compaore B., Mosse W., Seka E., Kietga G. (2020). Trichoblastic Carcinoma of the Upper Lip: A Case Report. Head Neck Cancer Res..

[110] Sajin M., Luchian M.C., Hodorogea Prisăcaru A., Dumitru A., Pătraşcu O.M., Costache D., Dumitrescu D., Oproiu A.M., Simionescu O., Costache M. (2014). Trichilemmal carcinoma—A rare cutaneous malignancy: Report of two cases. Rom. J. Morphol. Embryol..

